# Development of brain ventricular system

**DOI:** 10.1007/s00018-017-2605-y

**Published:** 2017-08-05

**Authors:** Vladimir Korzh

**Affiliations:** International Institute of Molecular and Cell Biology, Warsaw, Poland

**Keywords:** Brain ventricle, Circumventricular organs, Embryonic cerebrospinal fluid, Hydrocephalus, Voltage-gated K channel, Slit-ventricle syndrome

## Abstract

The brain ventricular system (BVS) consists of brain ventricles and channels connecting ventricles filled with cerebrospinal fluid (CSF). The disturbance of CSF flow has been linked to neurodegenerative disease including hydrocephalus, which manifests itself as an abnormal expansion of BVS. This relatively common developmental disorder has been observed in human and domesticated animals and linked to functional deficiency of various cells lineages facing BVS, including the choroid plexus or ependymal cells that generate CSF or the ciliated cells that cilia beating generates CSF flow. To understand the underlying causes of hydrocephalus, several animal models were developed, including rodents (mice, rat, and hamster) and zebrafish. At another side of a spectrum of BVS anomalies there is the “slit-ventricle” syndrome, which develops due to insufficient inflation of BVS. Recent advances in functional genetics of zebrafish brought to light novel genetic elements involved in development of BVS and circulation of CSF. This review aims to reveal common elements of morphologically different BVS of zebrafish as a typical representative of teleosts and other vertebrates and illustrate useful features of the zebrafish model for studies of BVS. Along this line, recent analyses of the two novel zebrafish mutants affecting different subunits of the potassium voltage-gated channels allowed to emphasize an important functional convergence of the evolutionarily conserved elements of protein transport essential for BVS development, which were revealed by the zebrafish and mouse studies.

## Introduction

The brain ventricular system (BVS) consists of brain ventricles and channels that connect ventricles. In mammals the BVS (from anterior to posterior) consists of two lateral (telencephalic) ventricles that via the intraventricular foramina (Monro) connect to the IIIrd (diencephalic) ventricle, which in turn via the sylvius aqueduct connects to the IVth (rhombencephalic or hindbrain) ventricle from where apertures (Lushka and Magendie) connect to subarachnoid space. In other species the BVS is less well understood. Similar to mammals, amphibians and birds have four brain ventricles each of which contains the choroid plexus (CP) [37, 58, 80].

Traditionally due to absence of means to observe events taking place behind the skull, progress in understanding the BVS relied on indirect or invasive studies of cerebrospinal fluid (CSF) flow. Hence it comes as no surprise that diseases affecting the BVS such as hydrocephalus often result from abnormal circulation of CSF and affect development of brain ventricles [44, 82]. A significant improvement of noninvasive analysis owing to a wider usage of functional magnetic resonance imaging (fMRI) has led to the discovery of intracranial pulsations linked to periodic changes in blood pressure and understanding that CSF flow depends upon systemic circulation. This emphasizes a role of a heart driving circulation, and tissues acting as absorbers of intracranial pulsation [42]. Mechanistically, CSF flow in the developing BVS is driven by a triad of factors—CSF production, beating cilia, and, depending on species, the Reissner fiber (RF) or its soluble components [26, 32, 34, 37, 50, 56, 70]. Studies in developmental neurobiology showed that an early developmental abnormality of BVS could be a cause of hydrocephalus and focused attention on ventricular epithelium (ependyma) [22]. Changes of ependymal integrity cause increase of cell proliferation and generation of eCSF resulting in hydrocephalus [9]. At the opposite end, reduction of cell proliferation and/or generation of eCSF may lead to intracranial hypotension and reduction of BVS reminiscent of over-drained ventricular shunt patients (slit-ventricle syndrome; [68] and microcephaly.

**Fig. 1:**
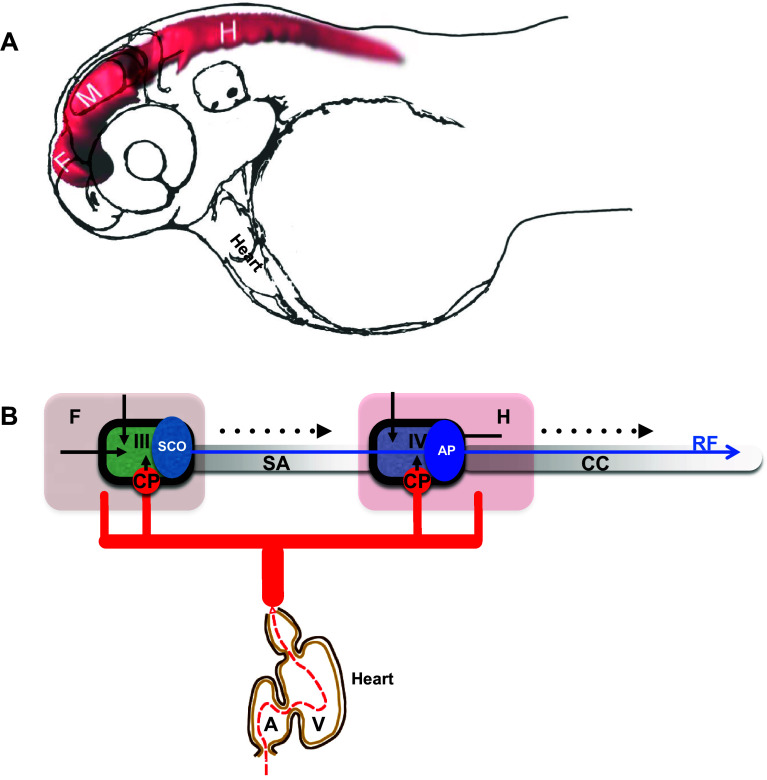
Development of the brain ventricular system of zebrafish. **a** The dye-filled ventricular system of 48 hpf zebrafish consists of three distinct cavities, which are reshaped as two ventricles and sylvius aqueduct connecting these. **b** Hypothetical scheme connecting the zebrafish blood circulation and the brain ventricular systems. During development influx of eCSF inflates and shapes the brain ventricular system [50, 51]. The choroid plexi contribute CSF enriched by ions, micronutrients and proteins. The SCO produces substances of the Reissner fiber, etc. *A* atrium, *AP* area postrema, *CC* central canal, *CP* choroid plexus, *F* forebrain, *H* hindbrain, *M* midbrain, *SA* Sylvius aqueduct, *SCO* subcommissural organ, *V* heart ventricle, *III* III brain ventricle, *IV* IV brain ventricle. *Black arrow* indicates embryonic CSF influx, *arrowhead* indicates CSF influx, *broken line* indicates direction of CSF flow. *Blue arrow* indicates Reissner fiber, *red solid lines* indicates vasculature and *black solid line* indicates ependyma

Development of BVS has been addressed to some extent. Relatively recent introduction of zebrafish into these studies allowed to study normal development of BVS in real time in vivo with high 3D resolution and its changes under experimental conditions [26, 32, 50, 70] (Fig. 1a). In contrast, its function during adulthood could be of interest in evolutionary perspective due to major morphogenetic adaptation of aquatic animals during transition to the four-chambered heart, terrestrial habitat and bipedal mode of locomotion, which probably caused major changes in distribution of bodily fluids and blood circulation. Currently, these processes remain not fully understood.

An organization of the neuroepithelial layer lining BVS is of significant interest. By generating CSF and forming a barrier that regulates intracranial pressure, it plays an important role in the development of BVS and its pathology. The specialized functions of the neuroepithelial barrier suggest its specialized organization, including the formation of developmental stage-specific tight junctions containing Claudin5a [3]. In support of this view, during mammalian development the neuroepithelial barrier of ventricular wall develops “strap” junctions limiting transport of biological molecules between BVS and deep cells of neural tube wall. This specialized organization may depend on developmentally restricted expression in ependyma of ECM proteins, namely, Claudin5 as well as *N*-cadherin, and β-and α-catenins [79].

In view of the fact that studies in model animals revealed a number of genes which deficiency causes early developmental abnormality of BVS, an idea was put forward that the initial phase of formation of this system takes place independent of systemic circulation driven by the cardiac activity, i.e., without involvement of CSF produced by the CP [50, 51, 70, 79] (Fig. 1b). These views could be traced back to pioneering work of Pollay and Curl [66], who demonstrated that ependyma generates CSF, and Desmond and Jacobson [23], who, without discriminating effects of eCSF and CSF, suggested that an enlargement of brain cavity in chick embryos requires a temporarily restricted increase of CSF pressure in specific subdivisions of developing brain due to a transient closure of BVS channels. Furthermore, it is accepted that CSF is produced by ependyma as interstitial fluid [1, 18, 69]. At least for now the bioimaging-based analysis of CP development in zebrafish transgenics expressing green fluorescent protein (GFP) led to conclusion that CP is formed after the initial phase of BVS formation [10, 11, 30]. These observations reveal developmental events as demonstrated by accumulation of cytosolic GFP in cells forming the CP primordium—*tela choroidea*. It is known that accumulation and maturation of GFP takes at least a couple of hours. Detection of GFP expression in *t. choroidea* depends on timing of initiation of expression of genes tagged by transposon insertion [30] such as, *sulf1* encoding Sulfatase 1, which modulates signaling by heparin-binding growth factors, including FGF2 [20, 47]. Sulf1 deficiency has been linked to microcephaly and cerebral dysfunction [21]. The patterns of GFP expression revealed by conventional confocal microscopy probably illustrate molecular events taking place somewhat earlier. Therefore, it is reasonable to expect that the secretion of CSF may start prior to the detection of GFP in the *t. choroidea*, perhaps, as early as the beginning of BVS inflation. To accurately address this matter, the specific time point of activation of CP function will need to be re-assessed using much more sensitive and developmental stage-specific bioimaging techniques. Therefore, it is important for the zebrafish BVS anatomy to be characterized in as much detail as that of mammals in order for comparative analysis to become possible.

### Neuroanatomy of the brain ventricular system in zebrafish

Zebrafish (*Danio rerio*) represents teleosts (bony fish) and belongs to the family *Cyprinidae*, second largest family of freshwater fish of this planet. Development of its relatively large, semi-transparent, and externally fertilized embryos takes about 52–55 h at 28.5 °C outside maternal organism and is easy to monitor under dissecting microscope. These features make the zebrafish embryo a good teaching tool easy to manipulate even in a small laboratory. Given a large collection of transgenics expressing fluorescent proteins in tissue-specific manner and mutants affecting development of various cell lineages, tissues or organs, the zebrafish could be very useful model animal to study development of BVS. Description of BVS in zebrafish is far from being satisfactory. Part of the problem is that comparing to mammals a different number of brain cavities is formed during development and their shape changes rather significantly. There were attempts to name these according to their position within major subdivisions of brain (telencephalon, diencephalon, midbrain, and hindbrain) [26, 77]. Their connection to brain ventricles is not evaluated with respect to other established landmarks of the brain. The circumventricular organs (CVO) occupy characteristic and well conserved in evolution positions in BVS. In mammals, nine CVOs are commonly recognized: the pineal gland (PIN), subfornical organ (SFO), organum vasculosum laminae terminalis (OVLT), paraventricular organ (PVO), median eminence (ME), neurohypophysis (NH), subcommissural organ (SCO), area postrema (AP) and CP [43]. These organs found mostly along the brain midline in vicinity of the IIIrd and IVth brain ventricles [25, 29, 45] with exception of CP of lateral ventricles of telencephalon. A vast study described about 18 different CVOs in 31 species belonging to various groups of vertebrates, from cyclostomes to mammals with the NH, ME, SCO and PIN found in almost all vertebrate species examined [75, 76]. In the zebrafish some CVOs were studied, including CP of the IIIrd and IVth ventricle [10, 11, 30], PIN [55], PP [19], NH and ME [8], SCO [27], AP [53]. Several of CVOs including CP express GFP in zebrafish transgenics ET33-E20 (Gateways; [30, 31].

**Fig. 2:**
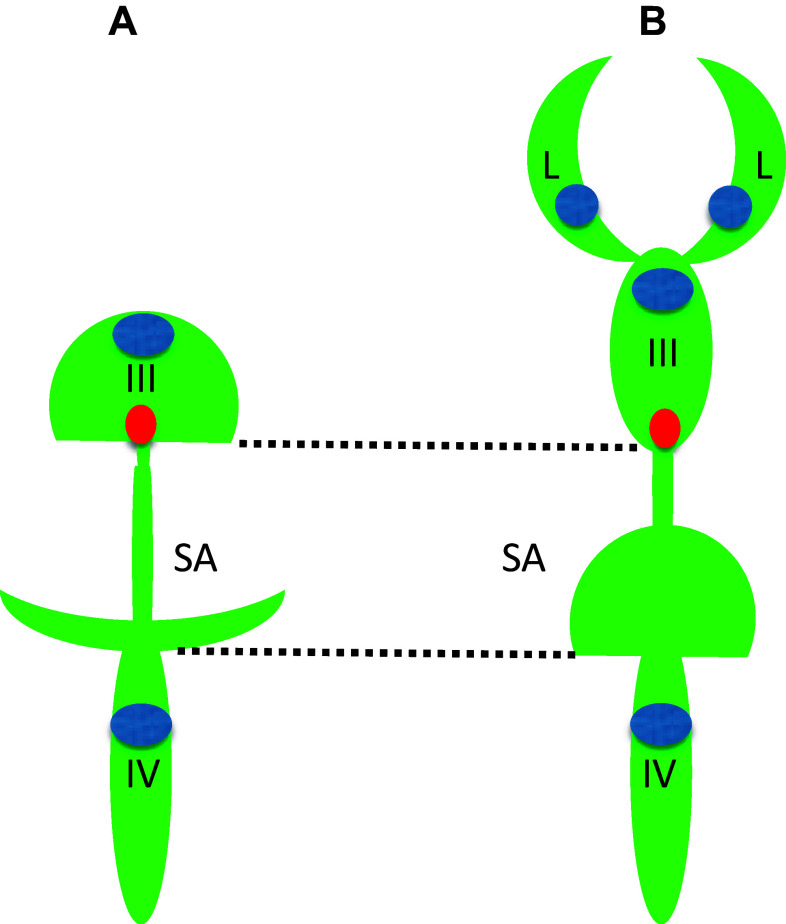
Comparison of brain ventricular system in 6 dpf zebrafish (**a**) and stage 53 *Xenopus* (**b**). The zebrafish larval BVS contains two ventricles unlike that of larval *Xenopus*. Whereas the IIIrd ventricle and posterior SA by the end of embryogenesis acquire rather complex configuration these are not that different from corresponding part of BVS in *Xenopus*. *Green*—ventricular system, *blue*—choroid plexus, *red*—subcommissural organ, *III*—IIIrd ventricle, *IV*—IVth ventricle, *L*—lateral ventricle, *SA*—Sylvius aqueduct (modified from Turner et al. [77], and Mogi et al. [58], correspondingly)

Unlike the telencephalon in mammals, which develops by inflation, i.e., similar to other brain regions, the zebrafish telencephalon develops by eversion. This means that compared to mammals, there is a significant morphological rearrangement along the mediolateral axis of the telencephalon. Importantly, in teleosts the telencephalon consists of a pair of solid lobes that seem to lack lateral ventricles. Nevertheless, the “telencephalic” ventricle was described in zebrafish [28, 62, 81]. It needs to be mentioned that this discussion was primarily concerned with details of reorganization of neural structures and not the BVS per se. And yet, during embryogenesis, at least three brain cavities develop in the zebrafish brain. This complicates classification of brain ventricles, so it needs to be sorted out based upon additional landmarks. All brain ventricles in mammals are found in specific neuroanatomical location and CP is a characteristic feature of all ventricles in all species [80]. At the level of forebrain only one CP was found at the midline of zebrafish brain [10, 11, 30]. This is consistent with an idea that the forebrain CP corresponds to the CPIII found in the diencephalon of other species. The cavity separating the telencephalon and diencephalon has been defined as “telencephalic” ventricle [28, 62] is associated with the CPIII, PIN-PP complex, SCO, ME and some other CVOs [31] and, thus, carries features of the IIIrd ventricle of other species. Interestingly, the dye-filling reveals well the dorsal part of the IIIrd ventricle, but not its ventral part [51, 70] (Fig. 1). This could be due to close apposition of the two apical surfaces of ventral brain similar to that in the spinal cord, where two apical surfaces closely appose each other dorsally [46, 61]. More posteriorly, a large transient cavity at the level of midbrain (sometime referred to as *optocoele*) does not contain CP or any other CVO. In mammals, the SCO is located at the posterior border of the IIIrd ventricle close to an entrance into the sylvius aqueduct (SA), which spans the midbrain and midbrain-hindbrain boundary (MHB) to connect to the IVth ventricle. In zebrafish, the SCO is located at the posterior border of IIIrd ventricle at the entrance to *optocoele*, which connects the IIIrd and IVth ventricles [27]. Thus, the *optocoele* most probably represents an early phase of the SA development. Similar to the spinal cord, the *optocoele* contains the midbrain roof plate, which while not being as extended along the dorsal–ventral axis as that of the spinal cord [46] nevertheless is elongated enough to be clearly defined. This illustrates a neuroanatomical element, which is common for the central canal and *optocoele*. Later in development the *optocoele* is much reduced due to significant expansion of the optic tectum dorsally and tegmentum ventrally. In parallel, its void acquires an intricate shape, which is more clearly manifested posteriorly [77]. Posterior to the MHB, the roof of hindbrain cavity contains the CPIV [11, 30] and the AP at the entrance to the central canal [31, 53]. These are clear landmarks of the IVth ventricle. Hence as a typical representative of teleosts, the zebrafish contains the BVS, which on one hand is less complex compared to that of mammals, i.e., two ventricles vs. four in mammals (Fig. 2). On the other hand some of its parts such as the IIIrd ventricle and posterior SA acquire rather complex configuration during development although its overall shape is not that different from that in *Xenopus*.

Given evolutionary conservation of molecular developmental mechanisms, the rules guiding the formation and function of BVS in zebrafish should well represent the basic molecular mechanism of BVS formation and function in mammals at least as far as ventricles III and IV are concerned. This is why the zebrafish is being used currently to study development and genetics of BVS. The zebrafish as a model animal provides a possibility to apply approaches of direct genetics, such as medium- to large-scale mutagenesis screens and bioimaging of the developing BVS in vivo. Still, it is possible that due to significant evolutionary distance between teleosts and mammals, some genes and/or gene functions with a role in the BVS development in teleosts were acquired due to an extra round of genome duplication in teleosts or lost or modified significantly during evolution of land animals and transition from aquatic environment to a terrestrial one, or in evolution of human during transition to bipedal mode of locomotion and vertical body posture. Hence more studies are requires to address developmental and evolutional diversity of the molecular machinery driving formation and function of BVS in zebrafish.

### Developmental genetics of the brain ventricular system

At the end of 2016 the term “abnormal brain ventricular system morphology” was associated with 549 mice mutants (http://www.informatics.jax.org/searchtool/Search.do?query=brain+ventricle&submit=Quick+Search) with 183 mutants showing enlarged brain ventricles and only ten with reduced brain ventricles. This suggested that the diagnosis of BVS deficiencies still largely relies on detection of gross morphological abnormality. Perhaps, it is easier to detect hydrocephalus compared to reduction of brain ventricles. Along the same line, given a connection of the reduction of brain ventricles and microcephaly and the fact that a similar search for the term “microcephaly” brought about 143 entries, a number of mutants with reduced brain ventricles could be underestimated. To add to that, hydrocephalus might be caused by defects in evolutionary conserved (in teleosts and mammals) components of signaling pathways, in particular those involved in planar cell polarity and ciliogenesis [65, 73]. Not all ependymal cells are ciliated and at least in mammals a significant proportion of these are tanycytes expressing a variety of tight junction proteins [2, 48, 60, 71]. This makes it possible that defects of BVS are intricately linked to defects of tissue integrity.

Despite limitations of current knowledge brought about by technical challenges, experimental evidence obtained due to analysis of zebrafish mutants occasionally allows to combine results obtained in different areas of science and develop a novel perspective. This concerns the formation of BVS also. During the large-scale mutagenesis screens of the 1990s, several mutants with deficient BVS, including *nagie oko* (*nok*), which affects *mpp5a* encoding the membrane protein, palmitoylated 5a (MAGUK p55 subfamily member 5 [54, 78], and *snakehead* (*snk*), which affects *atp1a1a.1* [40, 51] were found. It took several years to map these mutants and reveal their true potential for studies of the developing BVS. The *nok* mutants are characterized by abnormal brain neuroepithelium with no clear midline and disrupted junctional protein expression. They fail to undergo ventricle morphogenesis in a process dependent on their interaction of Mpp5a with Crumbs2a (Crb2a), which failure affects the transport of proteins of the apical complex [12, 17, 51]. Similar phenotype was described in the *pard6γβ* mutant [46, 61, 63]. In contrast, analysis of *snk* mutants with impaired BVS inflation revealed a complex role of Atp1α1a during formation of cohesive neuroepithelium, restriction of neuroepithelial permeability and production of CSF [17, 51].

This led to an understanding that a systematic classification of collection of zebrafish mutants exhibiting ventricle deficiency assayed using ventricular dye-filling, bioimaging and immunohistochemistry could be instrumental in defining the different stages of BVS development. As a result, four phenotypically distinct classes of mutants with early to late developmental defects ranging from abnormalities of epithelial junction and ventricle expansion to defects of MHB and ventricle lumen expansion were defined [50]. For example, detailed analysis of mutants with deficient MHB demonstrated that its formation is not entirely dependent upon changes in intracranial pressure. Here the two phases of this process were described. First includes a significant shortening of the MHB cells followed by the laminin-dependent basal constriction and apical expansion of a specialized group of MHB cells [35]. Second includes epithelial relaxation involving the myosin phosphatase regulator Mypt1 that regulates changes in cell shape during stretching of neuroepithelium. It is required for expansion of ventricular lumen [36]. Thus, formation of BVS is based upon a complex network of evolutionary conserved molecular events that could be efficiently analyzed using zebrafish mutants.

Despite being instrumental, mutants derived from the large-scale chemical mutagenesis were not easy to map. Therefore, there is a group of unmapped mutants relevant for studies of BVS, whose analysis is limited by the lack of knowledge of genes affected—*zon*, *all*, *fun* [4, 49, 82]. This situation may change as mapping of mutants is getting less challenging. Similarly, genes and/or mutants (e.g., Table 1) will be re-evaluated in future studies using other functional assays and their classification may change. There is also a group of insertional mutants that cause hydrocephalus. While these were mapped, they remain characterized insufficiently (*rps29*, *rpl7*, *rps12*, *sox32*, etc. [5]). Some of these mutants were linked to genes with a role in cell death or some other developmental events, which may not be relevant in the specific context of BVS development, whereas those linked to regulation in blood circulation could be rather useful. For these events to be better understood, further studies of such mutants are required. The availability of these research tools ready to be analyzed means that an entry threshold into this field is relatively low.

**Table 1:** Some zebrafish mutants with defects in the brain ventricular system

Gene	Zebrafish mutant/phenotype	Mice phenotype	Human phenotype	References
*mpp5a*	*nok/*reduced ventricle	No	Kabuki syndrome (microcephaly)	[12, 51]; http://www.malacards.org/search/results/mpp5
*prkci*	*has*/reduced ventricle	No	Huntington disease	[50]; http://www.malacards.org/search/results/prkci
*crb2*	*ome/*reduced ventricle	Open neural tube	Ventriculomegaly	[50]; http://www.malacards.org/search/results/crb2
*pard6γβ*	*pard6γβ/*reduced lumen	NA	NA	[46, 61]; http://omim.org/entry/608976?search=pard6g%20gene&highlight=pard6g%20gene
*med12*	*ott/*reduced ventricle	NA	Craniorachischisis, exencephaly	[50]; http://www.malacards.org/search/results/med12
*med14*	*log/*reduced ventricle	NA	NA	http://omim.org/entry/300182?search=med14&highlight=med14
*lamc1*	*sly/*reduced ventricle	E5.5 lethal	Occipital cephalocoele (?)	[50]; OMIM*150290
*lamb1*	*gap/*reduced ventricle	Embryonic lethal	Hydrocephalus	[50]; [67]
*atp1a1*	*snk*/reduced ventricle	Hypertension		[17, 51]; OMIM*182310
*kcnb1*	*kcnb1*/reduced ventricle	NA	Epileptic encephalopathy	[70, 72]; OMIM*600397
*fn1*	*nat/*reduced ventricle	Neuronal apoptosis		[50]
*sfpq*	*wis/*reduced ventricle	Renal dysfunction	NA	[50]; OMIM*605199
*kcng4b*	*kcng4b*/hydrocephalus	NA	NA	[70]

Some results obtained using methods of transient block and/or inhibition of gene activity probably illustrates incomplete suppression of gene activity and/or peculiarity of experimental procedure involved. For example, the targeted morpholino-mediated knockdown of either Atp1α3a or Atp1α3b leads to hydrocephalus, which authors interpreted as a result of imbalance of ion transport across plasma membrane resulting in excessive accumulation of CSF in the BVS [24]. In contrast, the analysis of the *atp1a3a* ^*tpl10*^ gene trap mutant zebrafish failed to show hydrocephalus in *atp1a3* ^*atpl10*^ homozygotes, but demonstrated larval lethality of *atp1a3a* ^*tpl10*^ homozygotes. This was interpreted in favor of an essential role for Atp1α3a in neural development and/or physiology [7], i.e., in concert with postembryonic lethality observed in the *atp1a3* mutant mice [59]. This example raises awareness that interpretation of results of the transient loss-of-function analysis should be done with caution. And yet one should keep in mind that a complete loss-of-function of gene activity may be a rare event in human population, where mutations detected could be, therefore, much less disruptive. Hence it is possible that the phenotype observed upon transient loss-of-function may to some extent represent the effect of more deleterious mutations. Alternatively, it is also possible that the effect of such experimental procedure is unspecific. These situations will need more experimental evidence to be analyzed before some of controversies could be resolved. At least for now a bulk of data links ATP1α3 to a range of neurological disorders, but fails to support brain ventricle dilation observed in zebrafish (http://omim.org/entry/182350).

Here it would be appropriate to remind about a large cohort of studies of the popular rodent model of hydrocephalus—*hyh* mutant mice [15, 41] and compare that with recent data obtained using the zebrafish model. In the latter a study of a role of the Kcnb1-Kcng4b axis in the BVS development revealed a rather satisfactory correlation of the mutant (stable) and morphant (transient) phenotypes as well as dominant-positive and –negative studies [70]. It was shown in mice that the BVS development is affected by the molecular impairment of the SNARE-mediated protein transport essential for regulated exocytosis of neurotransmitters, apical localization of proteins and determination of neuroepithelial cell fate. α-Snap is involved in a wide variety of membrane fusion events in eukaryotic cells, including the disassembly of the *cis*-SNARE complex and the target compartment, i.e., recycling and retrograde transport of components of *v*-SNAREs [39]. In the *hyh* mice a mutation of α-Snap results in delamination of neuroepithelial cells lining the BVS followed by hydrocephalus [16, 38]. In developing mice brain, proliferating cells in the ventricular zone (VZ) are found in two distinct regions: the dorsal walls of the IIIrd ventricle and SA. In hydrocephalic *hyh* mutants, the dorsal walls of the IIIrd ventricle and SA expand enormously, probably partially due to increased ependymogenesis [9]. A possibility exists that such increase of cell proliferation compensates for delamination of cells taking place earlier.

**Fig. 3:**
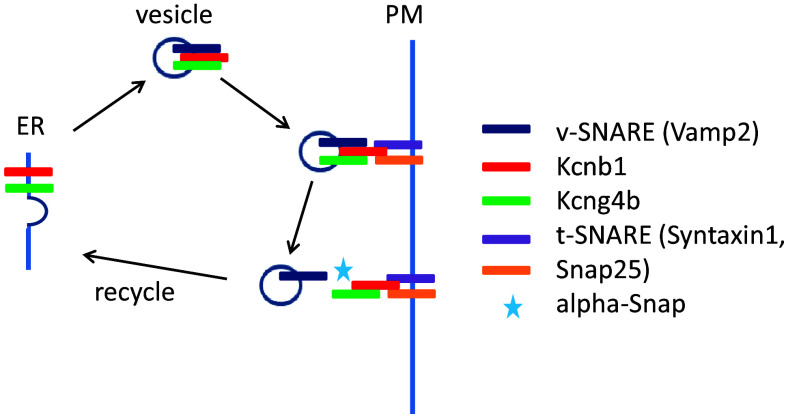
Genetic analyses reveal a role of the Kcnb1-Kcng4 axis in development of the BVS. Mutation/LOF of Kcg4b causes strong hydrocephalus and GOF—almost complete failure of ventricle inflation. In contrast, Kcnb1 mutant shows the slit-ventricle phenotype. The defect of Kcnb1 not only eliminates function of this protein as an electrically active subunit of voltage-gated Kv channel. Given its role in transport of Kcng4b, the defect of Kcnb1 negatively impacts function of both proteins, which counteract each other activity at the plasma membrane. Thus, the complete LOF of Kcnb1 could be partially compensated by a deficiency of Kcng4b transport. Note that Kcng4b is not expressed in adult animals suggesting that its modulation of Kcnb1 activity takes place only during development

Such scenario is very similar to that described recently in the zebrafish insertional mutant with aberrant splicing of the *kcng4b* mRNA, which encodes the silent subunit of the voltage-gated Kv channel—Kcng4 (or Kv6.4; [70]). It was shown by electrophysiologists that Kv6.4 functionally antagonizes electrically active subunit of the voltage-gated Kv channel—Kv2.1 (or Kcnb1; [13, 14, 64]). This mutation in Kcng4b may cause the dominant-negative peptide to be formed such as the one used to block the activity of Kv channel in vitro [6]. Unlike its homologue *kcng4a*, which is expressed in sensory cells, *kcng4b* is expressed in epithelial cells lining the cavities of BVS and other hollow organs, including otic vesicles as well as anterior and posterior eye chambers and lens. Similar to that in *hyh* mutants, when causing hydrocephalus *kcng4b* mutation first causes delamination of ependymal cells followed by their excessive proliferation. Similarly, events uncovered in this case could be traced to aberrant protein transport. To reach the plasma membrane, Kv6.4 interacts with Kv2.1, which transports Kv6.4 from ER to plasma membrane in a process of SNARE-mediated protein transport [14, 64]. Since developmental defects described for the *hyh* mice and Kcng4b (Kv6.4)-deficient zebrafish are very similar, it might be not too surprising that one of the outcomes of *hyh* mutation could be a deficient function of Kv channels due to abnormal protein transport. Indeed, it was shown already that one of *v*-SNAREs—VAMP2, interacts with Kv2.1 and inactivates it [52], whereas components of *t*-SNAREs—Syntaxin 1A and the Syntaxin/SNAP-25 complex bind directly to the Kv2.1 channel C-terminus [57, 74]. A disturbance of protein transport in *hyh* mutants with all probability will impact that of Kv2.1, which may explain the remarkable phenotypical similarity of phenotypes of the zebrafish and mice mutants (Fig. 3).

Taking into account counteracting activities of Kv6.4 and Kv2.1 established by electrophysiologists in vitro, it was gratifying to see that a mutation of *kcnb1* generated by site-specific mutagenesis caused a developmental phenotype reverse comparing to that of *kcng4b*, i.e., reduced cell proliferation and reduced BVS. Further support for functional antagonism of the two subunits of the Kv channel during BVS development was provided when over-expression of these two genes caused the phenotypes reverse to those in mutants [70]. Intriguingly, mutations of KCNB1 in human were linked to early infantile epileptic encephalopathy (OMIM*600397, OMIM#616056). Hence these findings revealed a common molecular denominator for two human hereditary diseases—epileptic encephalopathy and hydrocephalus. More broadly, it suggested that different mutations of KCNB1 may cause a wide range of hereditary diseases—a notion which certainly needs to be explored further. Recently, the epilepsy phenotype has been characterized in the zebrafish mutant of the Syntaxin-binding protein 1 (Stxbp1; [33]). Taken together, genetic and biochemical evidences available illustrate the functional link between the components of protein transport and BVS development in model animals and human.

## Conclusion

The zebrafish has emerged as a good animal model to study BVS development and various aspects of human hereditary diseases affecting BVS such as the slit-ventricle syndrome and hydrocephalus. Analysis of mice and zebrafish mutants provided different, but complementary views on a role of protein transport in physiology of ependymal cells lining fluid-filled organs such as brain ventricles. In view of expression of the same proteins in cells lining internal cavities of the ear and eye, it is expected that future studies will reveal that similar mechanisms dependent on voltage-gated K channel and interplay of its subunits operate in these organs too. This information is important for understanding molecular mechanisms of development of the fluid-filled organs and, in particular, integrity of neuroepithelial lining as well as pathology caused by its loss.
